# Texture Perception and Chewing of Agar Gel by People with Different Sensitivity to Hardness

**DOI:** 10.3390/gels11010005

**Published:** 2024-12-26

**Authors:** Vasily Smirnov, Daria Khramova, Elizaveta Chistiakova, Natalya Zueva, Fedor Vityazev, Inga Velskaya, Sergey Popov

**Affiliations:** Institute of Physiology of Federal Research Centre “Komi Science Centre of the Urals Branch of the Russian Academy of Sciences”, 50 Pervomaiskaya Str., 167982 Syktyvkar, Russia; smirnowich@yandex.ru (V.S.); dkhramova@gmail.com (D.K.); kvashninova.e@yandex.ru (E.C.); nata.zueva.2000@mail.ru (N.Z.); rodefex@mail.ru (F.V.); velskaya.psy@gmail.com (I.V.)

**Keywords:** food texture, agar gel, texture profile analysis, hardness, sensory analysis, hardness sensitivity, chewing function, salivation

## Abstract

Hardness is one of the dominant sensory characteristics of food. This study estimated the effect of sensitivity to hardness on the texture perception and chewing function using 2, 4, and 6% agar gels. Increasing the concentration of agar resulted in an increase in gel hardness and springiness, measured by texture profile analysis. Non-trained participants (*n* = 95) reported more subjective hardness and springiness during chewing gel samples as the agar concentration increased. Based on the relationship value of the instrumental and sensory data, all participants were divided into low-, medium-, and high-sensitivity groups (*n* = 25, 44, and 26). Low sensitivity to hardness was associated with low sensitivity to brittleness, springiness, chewiness, moisture, and swallowability. In all three groups, enhanced agar gel hardness increased the temporal chewing characteristics in a similar manner. However, in those with a high hardness sensitivity, the area amplitude of the masseter and temporalis muscles grew to a lesser extent than in those with a low or medium sensitivity. The activity of the suprahyoid muscles increased with the increasing agar gel hardness, regardless of sensitivity. All groups showed a similar salivation and bolus fragmentation while chewing gel. Thus, people’s sensitivity to hardness was associated with different perceptions of the gel’s textural properties and changes in masticatory muscle activity.

## 1. Introduction

Food gels have recently become much more popular in modern food technology due to their critical role in modern food design [1]. Food gels serve as thickeners and stabilizers because of their ability to retain a substantial amount of water while maintaining a solid-like structure [2], provide better protection and controlled release of bioactive components [3,4], and are used in the design of complex food shapes using three-dimensional (3D) printing [5]. The appropriate design of food gels can not only improve food quality but also improve the nutritional properties of foods replacing fats [6], increasing satiety by lowering food intake [7]. Food gels also play a critical role in structuring foods with desired sensorial textures [8].

Texture characteristics are typically classified into three categories: mechanical, geometrical, and other (product moisture and fat content) [9]. In this situation, five fundamental characteristics have been presented to describe the mechanical properties of foods: “hardness”, “cohesiveness”, “viscosity”, “springiness”, and “adhesiveness”. Although texture cannot be represented by a single characteristic but rather by a mixture of several, hardness is one of the most important features of food.

Firstly, food hardness is one of the common and dominant hedonic texture characteristics [10], and a large number of studies have shown that hardness affects the hedonic evaluation of model food gels [11,12] or gel-based food [13]. In a number of studies testing different gel-based foods, hedonic scores increased with increasing food hardness [14,15,16]. However, other studies have found a decrease in the acceptability of the food with increasing hardness, which may be due to decreased swallowing comfort and impaired flavor perception [17,18]. Secondly, more solid and hard food products have been associated with slower eating rates and extended oral exposure duration, which is expected to improve the sensory experience of eating, suggesting that hardness is a food quality that influences food intake [19]. The study [20] demonstrated that the eating rate is mostly determined by hardness rather than adhesiveness or stickiness. It is also believed that humans change their strategy of bolus formation depending on food texture via the perception of hardness as a primary feature [21]. Thirdly, the results of the recent systematic review showed that dietary hardness had a beneficial impact on behavior, cognition, and brain function in animals as well as humans [22]. These studies suggest the effect of hardness on chewing function, especially during the initial stages of a masticatory sequence [23]. And finally, developmental changes in food texture preferences demonstrate that hardness sensitivity may serve as a valid indicator of individual perceptivity. In general, as children grow older, their food texture preferences vary from soft to hard, which is understandable given that they develop teeth and chewing function [24].

The variability in oral physiology and oral processing obviously shapes individual texture sensitivity. The evidence that increased oral texture sensitivity is associated with food acceptability and choice was indirectly supported by a study by Zickgraf et al. [25], who showed that oral texture sensitivity is a major predictor of selective or picky eating in children and adults. Analyses of previous works [12,24,26,27,28,29,30] revealed that the relationship between texture (tactile) sensitivity and food perception or preference is uncertain and rather dependent on texture modality. In particular, acceptability of chocolate cream and yogurt decreased after particles were added to them only in highly sensitive to particles participants [27]. Lingual tactile sensitivity determined by von Frey filaments was not related to the overall pleasantness of yogurt [28], possibly due to a lack of relationship between methods for determining oral tactile sensitivity and the ability to recognize food hardness, as follows on from Shupe et al. [31].

The perception of texture properties and food acceptance also depend on the characteristics of oral processing. For example, the perception of sausage texture differed between people who chewed for short and long periods of time, since the middle of oral processing [32]. The ability to discriminate between jams with different viscosity and sugar content was higher in slow-chewing subjects compared to those who chewed fast [33]. The activity of chewing muscles was also strongly related to the perception of hardness [34]. The use of different mouth behavior influenced texture perception and preference [35]. In a study by Kim and Vickers [24], it was found that participants with a different saliva flow rate, chewing efficiency, biting force, and particle size sensitivity had unequal liking for textural properties. Therefore, it is reasonable to suggest that the variability in oral texture sensitivity, chewing function, and salivation in people leads to a different perception and food acceptance.

The aim of this study was to characterize the perception of agar gel texture and gel oral processing in people with different sensitivity to food hardness. To determine sensitivity to hardness, we used a previously proposed method [12], which, in our opinion, allows us to take into account the multi-parameter nature of texture related to oral physiology.

## 2. Results and Discussion

### 2.1. Instrumental Characterization of Agar Gels

**Figure 1:**
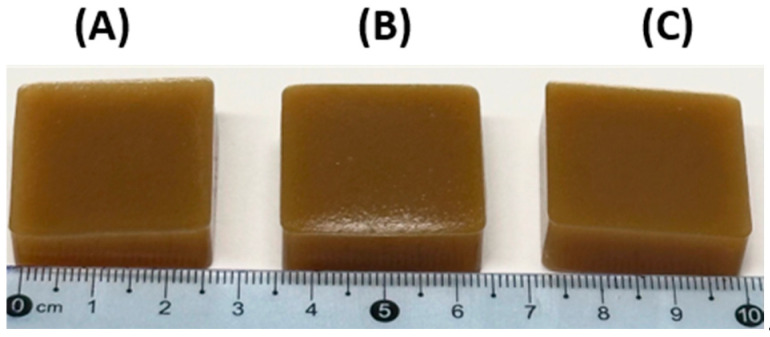
The appearance of agar gels A2 (**A**), A4 (**B**), and A6 (**C**) prepared with 2, 4, and 6% agar solutions.

Three model agar gels (A2, A4, and A6) were obtained by heating to 90 °C and cooling to 25 degrees Celsius 2, 4, and 6% agar solutions in peach juice with the addition of 50 g/L sugar. As can be seen from Figure 1, the obtained gel samples had the same brown color and the same shape in the form of a parallelepiped measuring (28 × 28 × 9 mm). The gels’ homogeneous appearance precluded the effect of appearance on people’s sensory perception.

Agar gels were of uniform density, but the water content decreased slightly with the increasing agar concentration (Table 1). The storage modulus G′ of the agar gels was greater than the loss modulus G″ throughout the linear viscoelastic region (LVE), indicating that it is a “viscoelastic gel” structure [36]. The G′_LVE_ and G″_LVE_ values increased with the increasing agar concentration. The elastic (k′) and loss (k″) moduli of the A4 and A6 gels were 4–6 times higher than those of the A2 gel.

The mechanical parameters of the A2, A4, and A6 gels were measured using texture profile analysis (TPA). Increasing the concentration of agar resulted in a significant increase in gel hardness, that is, the ability of the gel’s structure to withstand compression. The hardness of the A4 and A6 gel was 2.3 and 3.9 times higher than that of the A2 gel (Table 2). The cohesiveness, which reflects the intermolecular attraction by which the agar chains are held together, was the same for all samples. Springiness increased slightly with the increasing agar concentration, whereas the adhesiveness of the A4 and A6 gels, which reflects their ability to adhere to interfacial surfaces, was 50% higher than that of the A2 gel. Gumminess, measured as the product of hardness and cohesiveness, increased by 2.1 and 3.9 times in the A4 and A6 gels, respectively, when compared to the A2 gel (Table 2).

Analysis of the rheological and mechanical properties showed, as expected, that an increase in the agar concentration led to a strengthening of the gel. Agar is a polysaccharide composed of 3,6-anhydro-L-galactose and D-galactose units connected by α-(1→3) and β-(1→4) glycosidic linkages. At high temperatures, galactan chains assume a random and stiff coil shape. When the coils cool below the gelation temperature, they form helices and then aggregate, forming a gel, where hydrogen bonding is the key process. The rise in the gel hardness with the increased agar concentration was most likely caused by an increase in the number of cross-linking hydrogen bridges and the aggregation of double helices into a three-dimensional network [37]. Agar gels are classified as strong–soft–brittle materials according to the previously proposed three-dimensional model of the texture of solid foods [38].

Five primary parameters were proposed to present the mechanical properties of foods, including “hardness”, “cohesiveness”, “viscosity”, “springiness”, and “adhesiveness” [21]. Only three of them, hardness, adhesiveness, and viscosity, showed a significant rise with the agar concentration from 2 to 4%. A further increase in the agar concentration from 4 to 6% increased only the hardness and gumminess, the latter of which is a secondary TPA parameter. Therefore, it can be assumed that hardness, adhesiveness, and viscosity will have the greatest influence on the perception of the texture of agar gels by humans. Indeed, the mechanical hardness of the gels and the perception of hardness were highly correlated with each other (r = 0.72). However, this was not found for adhesiveness (r = −0.04), which was apparently due to the small change in this parameter with the increasing gel hardness (Table 2).

### 2.2. Liking and Perceived Texture of Agar Gels

A total of 95 respondents scored the overall and texture liking of the A2, A4, and A6 gels. The average score for the A2 gel was roughly 6 (“like slightly”), whereas the average score for the A6 gel was approximately 4 (“dislike slightly”), demonstrating a drop in overall like as the gel hardness increased due to an increase in the agar concentration. Similarly, increasing the hardness of the agar gel decreased its texture-liking score (Table 3). A high correlation coefficient (r = 0.88, *p* < 0.05) indicated a strong association of overall and textural liking as a result of the changes in gel hardness.

The perceived hardness and springiness of the samples increased with the increasing agar concentration, consistent with the changes in the instrumental hardness. The perceived hardness of the A4 and A6 gels was 3.1 and 4.4 times greater than that of the A2 gel (Table 4). The perceived springiness of the A4 and A6 gels was 47 and 84% greater than those of the A2 gel.

People perceived the gels with a higher agar content, the A4 and A6 gels, to be less brittle than the A2 gel. As the perceived hardness increased, so did the chewiness rates. The A4 and A6 gels had a significantly lower perceived moisture and swallowability compared to the A2 gels (Table 4). The Pearson correlation coefficient (r) demonstrated a moderate association of perceived hardness, chewiness, moisture, and swallowability with hedonic texture liking (Table 4).

Further regression analysis showed that swallowability and moisture contributed most to changes in the texture hedonic evaluation. The regression model, including only the most significant properties in terms of correlation (r > 0.30), was significant (F(4) = 28, *p* < 0.001, R^2^ = 0.29). The beta coefficients for swallowability and moisture were 0.26 (*p* < 0.001) and 0.21 (*p* = 0.002). Chewiness and hardness did not demonstrate a significant association with changes in texture liking (β = −0.13 and −0.07, *p* > 0.05).

The obtained data indicate a high sensitivity of humans to changes in the mechanical and rheological properties of the gel caused by an increase in the agar concentration from 4 to 6%. People perceived the springiness of the A6 gel to be higher than that of the A4, despite the fact that the instrumental analysis revealed the same springiness values for A6 and A4 (see Table 2). People’s remarkable sensitivity to the qualities of agar gel could be attributed to the dynamic nature of texture perception. It is generally accepted that different textural attributes are perceived at different stages of the sensory procedure, from the first bite through complete mastication and swallowing [9]. Hardness, brittleness, and viscosity are perceived during the first bite; chewiness and adhesiveness are perceived during chewing; and moisture and ease of swallowing are important in influencing the formation of a food bolus ready for swallowing.

A considerable amount of evidence was accumulated showing that food hardness affects overall liking. Our findings support the hypothesis that hedonic appraisal diminishes as the hardness of food gels increases [11,12]. However, there are conflicting data suggesting that food acceptance is conversely increased [14,15,16] or independent of food hardness [39,40,41].

### 2.3. Chewing Characteristics of Agar Gels

The temporal and amplitude electromyography (EMG) parameters of chewing the A2, A4, and A6 gels increased in accordance with the increase in their hardness (Table 5). Chewing the A4 and A6 gels required 50 and 79% more time than the A2 gel. The increase in chewing time was due to the increase in the chewing cycle number, while the chewing cycle time was the same for all agar gels. The masseter muscle’s amplitude parameters rose more significantly with the increasing gel hardness than did the temporalis and suprahyoid muscles. Thus, the area amplitude of the masseter muscle, which represents the muscle’s work across the full period of chewing the A4 and A6 gels, was 75 and 128% higher than when chewing the A2 gel. The work of the temporalis muscle during chewing of the A4 and A6 gels was 63 and 111% greater than that of the A2 gel, while the work of the suprahyoid muscles during chewing of the A4 and A6 gels was only 47 and 81% greater than that of the A2 gel. The activity index, which measures the muscle activity ratio, showed that the masseter muscle was more involved in chewing as the agar gel hardened.

The data support the hypothesis that the method of bolus generation varies according to the food texture, with the perception of hardness being a key factor. It is well established that increasing the food hardness increases the muscular activity during chewing, prolongs oral processing, and increases the number of chews required to prepare food for swallowing [42]. Chewing solid meals with a soft texture, such as agar gel, continues until the tension between the tongue and the hard palate during bolus squeezing is adequately decreased. However, prolonging the duration of oral processing did not improve the swallowability of the A6 gel. This may be owing to the gel’s low water content and perceived moisture rating.

### 2.4. Clustering of Hardness Sensitivity

**Figure 2:**
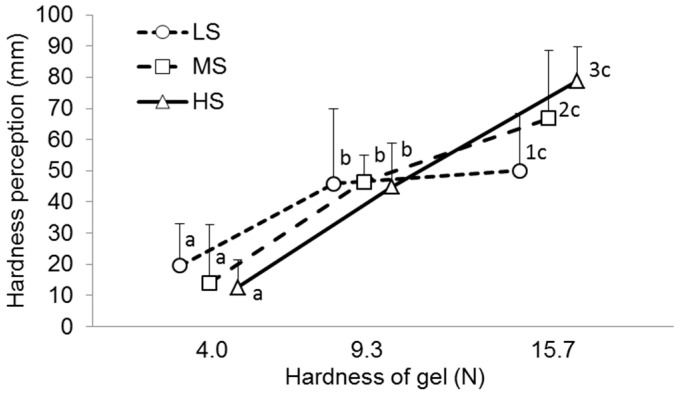
Perceived hardness of A2 (hardness—4.0 N), A4 (hardness—9.3 N), and A4 gels (hardness—15.7 N) in low (LS)-, medium (MS)-, and high-sensitivity (LS) groups. Different letters (numbers) mean significant difference between gels (groups) at *p* < 0.05. M ± SD.

The statistical technique previously provided by Puleo et al. [12] was used to compare the objective and sensory hardness data for the A2, A4, and A6 gels. The hardness scores of each participant were fitted with a linear equation for instrumental hardness, estimating both the angular coefficient and the R^2^ coefficient of the curve and utilizing them as clustering variables. Participants were divided into three groups based on the slope and R^2^ value of the linear equation, and their ability to determine gel hardness is presented in Figure 2.

The median slope and R^2^ values were 4.46 and 0.94 for the entire group (n = 95). Twenty-six participants whose scores correlated to a linear equation with both a high slope (>4.46) and high R^2^ coefficient (>0.94) were included in the high-sensitivity group (HS group) since they had a very high ability to discriminate hardness differences among samples. Twenty-five participants whose scores correlated to a linear equation with both a low slope (<4.46) and low R^2^ coefficient (<0.94) were included in the low-sensitivity group (LS group) since they had a poor ability to discriminate hardness differences among samples. The medium-sensitivity group (MS group, n = 44) included participants who had both linear equation parameters with different values relative to the median.

### 2.5. Sensory Evaluation of Agar Gels in Groups of Different Hardness Sensitivity

**Figure 3:**
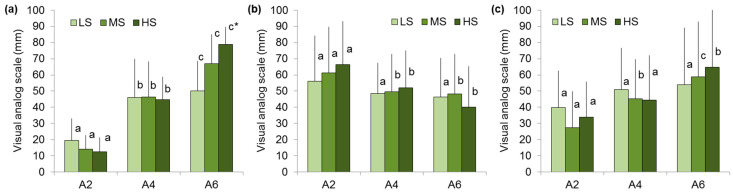
Hardness (**a**), brittleness (**b**), and springiness (**c**) perception of A2, A4, and A6 gels in low (LS)-, medium (MS)-, and high (HS)-sensitivity groups. Different letters mean significant difference between gels and * in comparison with LS group at *p* < 0.05. M ± SD.

Participants in the LS group rated the hardness of the A4 gel as 2.3 times higher than the A2 gel. However, the hardness rating of the A6 gel was only 9% higher than the A4 gel, while the instrumental hardness of A6 exceeds A4 by 1.7 times. In the HS group, the A4 gel obtained hardness ratings 3.5 times higher than the A2 gel, and the hardness rating of the A6 gel was 1.8 times higher than the A4 gel, which corresponds to the change in the instrumental hardness (Figure 3a). It was discovered that low sensitivity to hardness was associated with low sensitivity to brittleness and springiness. In particular, participants in the LS group scored the brittleness of the A2, A4, and A6 gels as the same. Participants with a medium and high hardness sensitivity assessed the A4 gel as 18–21% less brittle than the A2 gel, while there was no difference in brittleness between A6 and A4 (Figure 3b). Participants in the LS group found the springiness of the A2, A4, and A6 gels to be similar. Participants in the MS and HS groups showed higher springiness ratings for the A4 and A6 gels than for the A2 gel (Figure 3c).

**Figure 4:**
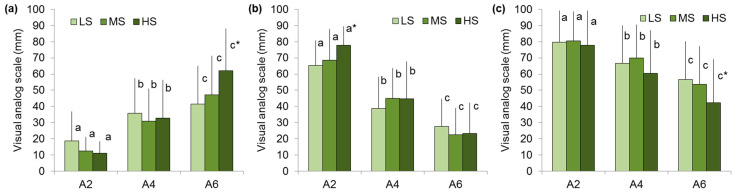
Chewiness (**a**), moisture (**b**), and swallowability (**c**) perception of A2, A4, and A6 gels in low (LS)-, medium (MS)-, and high (HS)-sensitivity groups. Different letters mean significant difference between gels and * in comparison with LS group at *p* < 0.05. M ± SD.

It was found that an increase in the instrumental and perceived hardness of the agar gels was associated with higher chewiness ratings in all groups. People with low, medium, and high sensitivity to hardness gave chewiness ratings for the A6 gel 2.2, 3.8, and 5.6 times higher than for the A2 gel (Figure 4a). The moisture scores of the A2 gel were higher in the HS group than the LS group (Figure 4b). The decrease in swallowability with increasing agar gel hardness is more pronounced in individuals with high sensitivity than in those with low sensitivity. In particular, the bolus swallowability scores were reduced by 46 and 29% in the HS and LS groups, respectively (Figure 4c).

**Figure 5:**
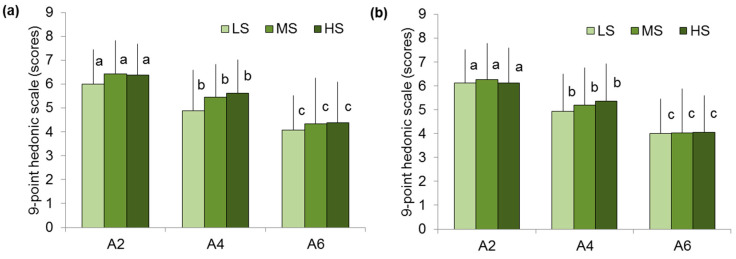
Overall liking (**a**) and texture liking (**b**) of A2, A4, and A6 gels in low (LS)-, medium (MS)-, and high (HS)-sensitivity groups. Different letters mean significant difference between gels at *p* < 0.05. M ± SD.

Overall and texture-like scores were similar among the LS, MS, and HS groups (Figure 5).

### 2.6. Chewing Parameters in Groups of Different Hardness Sensitivity

The temporal characteristics of chewing were enhanced with the increasing agar gel hardness in all three groups: LS, MS, and HS. The variations in the amplitude characteristics of the masseter and temporalis muscles due to the increasing agar gel hardness differed between the HS, LS, and MS groups (Table 6). The maximal EMG amplitude of the masseter muscle in the LS and MS groups was 29–30 and 44–47% higher when chewing the A4 and A6 gels compared to the A2 gel. Among participants with a high hardness sensitivity, the maximum amplitude of the masseter muscle was 18 and 27% higher when chewing the A4 and A6 gels compared to the A2 gel. As the agar gel hardness rose, the area amplitude of the masseter muscle increased to a smaller extent in people with a high than low and medium hardness sensitivity.

A similar pattern was found for the amplitude characteristics of the temporalis muscle, which increased less in the HS than in the LS and MS groups (Table 6). The activity of the suprahyoid muscles was enhanced with the increasing agar gel hardness, regardless of the subject’s sensitivity to hardness.

Salivation and bolus fragmentation during chewing the A6 gel were similar in the LS, MS, and HS groups (Table 7).

Chewing activity, salivation, and chewing efficiency are among the physiological factors that drive the perception of food texture [24]. The results obtained show that chewing effectiveness, as evaluated by particle size reduction, and saliva flow rate are the same in people with varying sensitivity to hardness, despite variances in masticatory muscle activity. It can be assumed that differences in the work of the masticatory muscles may be due to the psychological or sociocultural characteristics of people with a high sensitivity to hardness. However, it should also be noted that the actual state of the participant’s teeth was not assessed here, which is a limitation of this study. It is obvious that the actual state of the teeth is an important factor that determines the perception of food texture and hardness in particular. It is possible that tooth condition caused differences in masticatory muscle activity and hardness sensitivity

Previously, Puleo et al. [12] showed that young subjects are more sensitive to hardness than adults, and males more so than females. The present study confirmed Puleo’s findings that a significant difference is observed between the individuals in terms of perceived hardness. In addition, we showed that individuals with a high sensitivity to hardness had increased sensitivity to gel brittleness and springiness. Furthermore, the increase in sensitivity to hardness was accompanied by a greater decrease in swallowability with the increasing gel hardness. Identifying the factors that determine the individual perception of food texture is clearly necessary for the further understanding of texture preferences and food choice among people.

## 3. Conclusions

Thus, 2, 4, and 6% agar gels were prepared to characterize the texture perception and oral processing in people with a different sensitivity to the hardness of food with a solid–soft–brittle structure. Increasing the concentration of agar resulted in an increase in gel hardness and springiness, measured by texture profile analysis. The strengthening of the gel was revealed by rheological analysis, which showed a significant increase in the storage modulus G′ and the loss modulus G″ with the increasing agar concentration. Non-trained participants reported more subjective hardness and springiness during chewing of the gel samples as the agar concentration increased, which corresponded to changes in the objective hardness. A decrease in the overall and texture liking was found as the gel hardness increased, probably due to a decrease in the gel moisture and swallowability. The comparison of the instrumental and sensory hardness data for agar gels revealed three clusters of people: those with low, medium, and high sensitivity to hardness. High sensitivity to hardness was linked to increased sensitivity to gel brittleness and springiness. Furthermore, when the agar gel hardness increased, swallowability decreased more evidently in persons with high sensitivity than in those with low sensitivity. Unusual texture sensitivity of people in the high-sensitivity group was accompanied by a decrease in the degree of change in the amplitude of the masticatory muscles with an increase in the objective gel hardness. No effect of sensitivity to hardness on the hedonic perception of agar gels was found. Possible physiological consequences of the differences in hardness sensitivity remain to be clarified.

## 4. Materials and Methods

### 4.1. Preparation and Characterization of Gels

In this study, a model food gel was used, the hardness of which was changed by varying the concentration of agar. Agar powder (Zhenpai Hydrocolloids Co., Ltd. Zhangzhou City, Fujian Province, China) had a moisture content of 7.97 wt%, pH 6.46, and 1.5% gel strength of 1150 g/cm^2^ as was taken from the manufacturer’s certificate. Gels were prepared as follows. Agar solutions (2, 4, and 6 wt%) containing sugar (50 g/L) and freeze-dried peach (50 g/L, Stoing, Ltd., Saint Petersburg, Russia) were heated to 90 °C and cooled to 25 °C. Samples were covered by plastic wrap and stored at 4 °C for the night (14–15 hs). Mechanical and rheological properties of the gels, as well as hardness perception by participants, were performed the next day.

The weight of the gel samples was measured with an accuracy of 0.1 mg (AG245, Mettler Toledo International, Greifensee, Switzerland) to calculate the density as weight/volume [43]. The water content was determined by the gravimetric method by comparing the weight of the reference gel before and after drying [44]. The pH of gels was determined using the SevenEasy S20 pH meter (Mettler-Toledo GmbH, Schwerzenbach, Switzerland) after they were homogenized in water at a 1:10 ratio [45]. Three repeats were performed.

The rheological measurements were made with a rotational-type rheometer (Anton Paar, Physica MCR 302, Graz, Austria) fitted with a parallel plate geometry (diameter 25 mm; clearance 4.0 mm) used for the strain and frequency sweep measurements as described by a previous study [43].

The mechanical properties of the gels were determined by the double compression test method as was described earlier [44]. In brief, two gel samples (9 × 9 × 9 mm) were taken from each batch and double compressed at 25 °C using a cylindrical aluminum P/25 probe (25 mm diameter). The pre- and post-test speeds were 5.0 mm/s, and the test speed was 1 mm/s until a 100% strain. Eight measurements were made on each gel type. Five parameters of texture (hardness, cohesiveness, springiness, adhesiveness, and gumminess) were determined from the obtained force–time graph by Texture Exponent 6.1.4.0 software (Stable Micro Systems Ltd., Godalming, UK).

### 4.2. Participants

The invitation to participate was distributed to scientific staff and university students by announcement and e-mail. Inclusion criteria were age from 18 to 45 years, male and female, and desire to participate in the study confirmed by informed consent. Exclusion criteria were food restriction, food allergy, acute inflammatory diseases, medication, pregnancy and lactation, mastication dysfunction, and dental treatments [31,39], about which participants were asked verbally. A total of 109 study participants were recruited, of whom 14 were excluded from the analysis because they could not correctly recognize the hardness of the gels (as described in Section 4.4). Thus, 95 participants (62% women), whose mean age and BMI were 31.4 (SD = 8.2) years and 24.6 (SD = 4.0) kg/m^2^, respectively, were included in the study.

All participants were given detailed instructions on how to conduct the survey. They then signed informed consent and completed a participant questionnaire in which they indicated their gender, age, height, weight, and whether they visited a dentist in the last week. Additionally, they assessed subjective hunger and fullness using a 100 mm visual analog scale to control their satiety level [42]. The study protocol was approved by the Bioethics Committee of the Institute physiology of Federal Research Centre “Komi Science Centre of the Urals Branch of the Russian Academy of Sciences” (approval no. 10/10 March 2022). At the end of the study, each participant received compensation for time spent.

### 4.3. Sensory Analysis and Gel Acceptability Determination

The participants underwent a single test lasting 30 min (from 11:00 to 13:00) in a sensory room in accordance with the requirements of ISO 8589 [46]. Testing included three sessions. In the first session, participants chewed the gels in comfort mode, signaling the start and end of chewing. EMG recordings were made at this time. Just after chewing, participants determined the swallowability perception of the sample and evaluated texture and overall liking. In the second session, they evaluated the sensory properties of the samples according to the following plan: flavor before chewing; hardness, brittleness, and moisture at the first chewing movements; springiness, adhesiveness, and chewiness at the full chewing cycle of the second piece of the sample. Definition and evaluation techniques for texture characteristics were based on the guidelines of ISO 11036 [47]. Sensory and hedonic scores were assessed using a 100 mm visual analog scale and a 9-point hedonic scale, respectively [48]. In the third session, participants chewed the samples comfortably to a swallow-ready state and then spit the food ball into a container to determine salivation and bolus fragmentation.

### 4.4. Hardness Sensitivity Determination

Food texture sensitivity was assessed using the method proposed by Puleo et al. [12,29] to distinguish consumers’ sensitivity levels to texture attributes. In our opinion, this method has a significant advantage over methods for determining tactile sensitivity that do not take into account the multimodal nature of texture. In agreement with this methodology, participants’ hardness perception scores were compared to the mechanical hardness of the samples, and a linear equation for each subject was constructed from the data. Fourteen participants showed insufficient ability to recognize the hardness of the gels as they had a low R^2^ coefficient value (below 0.70) and were therefore excluded from the analysis. The remaining participants (n = 95) were divided into three groups based on the slope angle and R^2^ value of the linear equation. The low- or high-sensitivity group included participants whose slope and R^2^ values were below or above the median value (slope = 4.46 and R^2^ = 0.94) calculated across the entire sample, respectively. The group with medium sensitivity included people whose slope and R^2^ values were different from the median. The following characteristics of participants in the three groups were similar: men-to-women ratio, mean age, body mass index, and feeling of hunger and fullness (*p* > 0.05).

### 4.5. Chewing Behavior

The activity of the masseter (masseter muscle), temporal muscles (temporalis muscle), and suprahyoid muscles (suprahyoid muscles) was determined by surface EMG recording using a Neuro-MEP system with 4-channel amplifiers (Neurosoft, Ivanovo, Russia), with subsequent visualization and data analysis in the Neuro-MEP.NET program (ver. 4.2.6.5). For this purpose, two pairs of disposable gel electrodes (F9040, Fiab, Italy) were attached on the preferred side in the center of the masseter muscle and in the projection of the anterior temporalis muscle. The electrodes (11 × 34 mm) were placed on the skin strictly symmetrically with an inter-electrode distance of 20 mm and fixed with a fixation patch. The skin was degreased at the attachment site to reduce resistance. The ground band electrode was applied to the wrist of the left hand. The following parameters were used in the analysis: chewing time (s), chewing cycle number (times), maximum amplitude value (μV), and area amplitude value (μV × s).

### 4.6. Bolus Characterization

Bolus samples obtained in the third session after chewing the A6 gel were used to determine salivation and bolus fragmentation. Salivation was determined gravimetrically in accordance with [49]. The participants were asked to chew a pre-weighed quantity (~4 g, 10 × 10 × 20 mm) of the sample in a single mouthful as naturally as possible and spit out the bolus just before swallowing for further analysis. Then, participants gently rinsed their mouth with water (10 g) and expectorated the remaining food particles into the same container. The weight of each wet bolus sample (accurate to 0.01 g) was calculated as the combined weight of expectorated material less the weight of rinsing water.

Bolus saliva uptake (%) is expressed as follows:

(wet bolus (g) − wet sample (g))/wet sample (g) × 100.(1)

The rate of saliva incorporation (g/min) was calculated as follows:

(wet bolus (g) − wet sample (g))/chewing time (min).(2)

Bolus fragmentation was measured by manual sieving according to the procedure described previously [50] with slight modification. After weighting, the bolus was first spilled onto a nylon cloth with a 1 mm mesh size (Russia) and mushy bolus pellets were freeze-dried for the dry sieving. Dried mushy bolus pellets were manually sieved through the stainless sieve of 1.6 mm mesh size (Russia) and the particles retained on the sieve were carefully weighed (accurate to 0.1 g), and the weight results were expressed as the weight percent (wt%) of the dry bolus weight.

### 4.7. Statistical Data Analysis

The data obtained were analyzed using Microsoft Excel software and the R-based Jamovi software (Version 2.3) [51]. All variables were checked for normality and homogeneity of variance by the Shapiro–Wilk and the Levene tests, respectively. Differences between the two groups were evaluated using the chi-square test and Mann–Whitney U-test for qualitative and quantitative data, respectively. Friedman and Kruskal–Wallis tests were used to determine differences between the three groups when comparing the A2, A4, and A6 gels and participants with low, medium, and high sensitivities, respectively. The relationship of quantitative data was evaluated using Pearson correlation as well as linear regression. Data were presented as mean ± standard deviation. Differences were considered significant at *p* < 0.05.

## Figures and Tables

**Table 1:** General and rheological characteristics of A2, A4, and A6 gels.

Parameter	Agar Gel		
	A2	A4	A6
Density (mg/mm^3^)	1.08 ± 0.03 ^a^	1.12 ± 0.07 ^a^	1.12 ± 0.06 ^a^
Water content (%)	85.1 ± 0.1 ^a^	82.5 ± 0.1 ^b^	79.9 ± 0.2 ^c^
pH	4.17 ± 0.03 ^a^	4.27 ± 0.05 ^b^	4.26 ± 0.02 ^b^
G’_LVE_ (Pa)	31,156 ± 1052 ^a^	133,519 ± 11,681 ^b^	168,248 ± 40,533 ^c^
G″_LVE_ (Pa)	2455 ± 1010 ^a^	15,506 ± 7665 ^b^	20,050 ± 13,911 ^c^
Tan [δ]_LVE_	0.080 ± 0.036 ^a^	0.120 ± 0.067 ^ab^	0.164 ± 0.079 ^b^
k’ (Pa·s)	35,720 ± 1455 ^a^	144,006 ± 11,551 ^b^	144,909 ± 113,361 ^b^
k″ (Pa·s)	1645 ± 94 ^a^	8477 ± 761 ^b^	10,529 ± 4519 ^b^
η·s	5605 ± 304 ^a^	22,426 ± 1481 ^b^	29,241 ± 15,693 ^b^
Viscosity K (Pa·s)	5605	24,143	22,948

M ± SD; different letters show differences among gels at *p* < 0.05 (n = 8). Storage modulus (G′LVE), loss modulus (G″LVE), and loss tangent (Tan [δ]LVE) as measured in the amplitude sweep test (1 Hz. 20 °C). The elastic (k′), loss (k″) moduli, and the slope of complex viscosity (η·S) as measured in the frequency dependence test (0.3 < ω < 60.0 Hz) of viscoelastic parameters.

**Table 2:** Mechanical properties of A2, A4, and A6 gels.

Parameter	Agar Gel		
	A2	A4	A6
Hardness (N)	4.03 ± 0.26 ^a^	9.35 ± 0.68 ^b^	15.71 ± 1.21 ^c^
Cohesiveness	0.25 ± 0.09 ^a^	0.24 ± 0.04 ^a^	0.25 ± 0.07 ^a^
Springiness	0.98 ± 0.05 ^a^	1.02 ± 0.08 ^ab^	1.04 ± 0.10 ^b^
Adhesiveness (N×s)	0.02 ± 0.00 ^a^	0.03 ± 0.01 ^b^	0.03 ± 0.01 ^b^
Gumminess	1.03 ± 0.46 ^a^	2.21 ± 0.37 ^b^	3.99 ± 1.17 ^c^

M ± SD; different letters show differences among gels at *p* < 0.05 (n = 8).

**Table 3:** Liking evaluation of A2, A4, and A6 gels using 9-point hedonic scale.

Parameter	Agar Gel		
	A2	A4	A6
Overall liking (score)	6.3 ± 1.4 ^a^	5.4 ± 1.5 ^b^	4.3 ± 1.7 ^c^
Texture liking (score)	6.2 ± 1.5 ^a^	5.2 ± 1.6 ^b^	4.0 ± 1.7 ^c^

M ± SD; different letters show differences among gels at *p* < 0.05 (n = 95).

**Table 4:** Texture evaluation of A2, A4, and A6 gels using 100 mm visual analog scale and relationship texture parameters with overall liking.

Parameter	Agar Gel			r with Overall Liking
	A2	A4	A6	
Hardness (mm)	15 ± 10 ^a^	46 ± 20 ^b^	66 ± 20 ^c^	−0.42 *
Brittleness (mm)	61 ± 28 ^a^	50 ± 22 ^b^	46 ± 25 ^b^	0.19 *
Springiness (mm)	32 ± 23 ^a^	47 ± 25 ^b^	59 ± 35 ^c^	−0.06
Adhesiveness (mm)	24 ± 20 ^a^	25 ± 19 ^a^	24 ± 22 ^a^	0.16 *
Chewiness (mm)	14 ± 12 ^a^	33 ± 21 ^b^	50 ± 26 ^c^	−0.40 *
Moisture (mm)	70 ± 17 ^a^	43 ± 20 ^b^	24 ± 17 ^c^	0.44 *
Swallowability (mm)	79 ± 19 ^a^	66 ± 23 ^b^	51 ± 25 ^c^	0.48 *

M ± SD; different letters show differences among gels at * *p* < 0.05 (n = 95).

**Table 5:** EMG temporal and amplitude characteristics during chewing A2, A4, and A6 gels.

Parameter	Gels		
	A2	A4	A6
Temporal characteristics			
Chewing time (s)	14 ± 8 ^a^	21 ± 10 ^b^	25 ± 12 ^c^
Chewing cycle number (times)	19 ± 8 ^a^	29 ± 12 ^b^	34 ± 14 ^c^
Chewing cycle time (ms)	754 ± 219 ^a^	723 ± 163 ^a^	745 ± 175 ^a^
Amplitude characteristics of masseter muscle			
Maximal amplitude (mcV)	719 ± 508 ^a^	910 ± 634 ^b^	1016 ± 713 ^c^
Mean amplitude (mcV)	25 ± 19 ^a^	32 ± 22 ^b^	36 ± 24 ^c^
Area amplitude (mV·ms)	415 ± 357 ^a^	725 ± 531 ^b^	945 ± 781 ^c^
Amplitude characteristics of temporalis muscle			
Maximal amplitude (mcV)	596 ± 409 ^a^	699 ± 436 ^b^	765 ± 468 ^c^
Mean amplitude (mcV)	21 ± 13 ^a^	25 ± 14 ^b^	28 ± 14 ^c^
Area amplitude (mV·ms)	358 ± 321 ^a^	586 ± 451 ^b^	758 ± 559 ^c^
Amplitude characteristics of suprahyoid muscles *			
Maximal amplitude (mcV)	668 ± 316 ^a^	782 ± 409 ^b^	825 ± 409 ^c^
Mean amplitude (mcV)	32 ± 14 ^a^	35 ± 15 ^b^	36 ± 15 ^c^
Area amplitude (mV·ms)	545 ± 319 ^a^	802 ± 474 ^b^	985 ± 628 ^c^
Activity index			
Masseter/temporalis m. (%)	7 ± 21 ^a^	10 ± 20 ^b^	10 ± 20 ^b^
Masseter/suprahyoid m. (%)	−15 ± 27 ^a^	−6 ± 27 ^b^	−3 ± 26 ^c^

M ± SD; different letters show differences among gels at *p* < 0.05 (n = 95; * n = 92).

**Table 6:** EMG temporal and amplitude characteristics during chewing A2, A4, and A6 gels in low (LS)-, medium (MS)-, and high-sensitivity (HS) groups.

Parameter	Sensitivity Group	Agar Gel		
		A2	A4	A6
Temporal characteristics				
Chewing cycle number (times)	LS	20 ± 7 ^a^	29 ± 11 ^b^	36 ± 14 ^c^
	MS	18 ± 6 ^a^	29 ± 12 ^b^	32 ± 13 ^c^
	HS	22 ± 9 ^a^	30 ± 14 ^b^	36 ± 17 ^c^
Chewing time (s)	LS	14.8 ± 7.4 ^a^	21.3 ± 9.2 ^b^	26.3 ± 10.8 ^c^
	MS	12.5 ± 5.1 ^a^	19.1 ± 7.9 ^b^	22.6 ± 8.6 ^c^
	HS	17 ± 11.7 ^a^	22.6 ± 14 ^b^	27.1 ± 17.8 ^c^
Amplitude characteristics of masseter muscle				
Maximal amplitude (mcV)	LS	774 ± 729 ^a^	997 ± 795 ^b^	1116 ± 988 ^b^
	MS	732 ± 452 ^a^	949 ± 583 ^b^	1076 ± 626 ^c^
	HS	646 ± 335 ^a^	762 ± 544 ^ab^	822 ± 514 ^b^
Area amplitude (mV·ms)	LS	432 ± 519 ^a^	733 ± 671 ^b^	1043 ± 1158 ^c^
	MS	416 ± 274 ^a^	770 ± 496 ^b^	968 ± 616 ^c^
	HS	398 ± 310 ^a^	641 ± 449 ^b^	816 ± 598 ^c^
Amplitude characteristics of temporalis muscle				
Maximal amplitude (mcV)	LS	584 ± 381 ^a^	686 ± 417 ^b^	789 ± 483 ^c^
	MS	608 ± 435 ^a^	752 ± 474 ^b^	800 ± 475 ^c^
	HS	588 ± 405 ^a^	621 ± 387 ^ab^	681 ± 451 ^b^
Area amplitude (mV·ms)	LS	353 ± 260 ^a^	586 ± 347 ^b^	813 ± 522 ^c^
	MS	349 ± 257 ^a^	610 ± 463 ^b^	780 ± 602 ^c^
	HS	379 ± 453 ^a^	544 ± 523 ^b^	670 ± 529 ^c^
Amplitude characteristics of suprahyoid muscles				
Maximal amplitude (mcV)	LS	743 ± 326 ^a^	873 ± 464 ^ab^	946 ± 444 ^b^
	MS	642 ± 302 ^a^	786 ± 400 ^b^	782 ± 371 ^b^
	HS	643 ± 329 ^a^	692 ± 362 ^a^	782 ± 426 ^b^
Area amplitude (mV·ms)	LS	600 ± 309 ^a^	867 ± 488 ^b^	1087 ± 519 ^c^
	MS	512 ± 281 ^a^	781 ± 429 ^b^	917 ± 531 ^c^
	HS	547 ± 386 ^a^	775 ± 540 ^b^	1000 ± 842 ^c^

M ± SD; different letters mean significant difference between gels at *p* < 0.05.

**Table 7:** Salivation and bolus fragmentation during chewing A6 gel in low (LS)-, medium (MS)-, and high-sensitivity (HS) groups.

Parameter	Sensitivity Group		
	LS	MS	HS
Saliva incorporation rate (g/min)	4.0 ± 3.2 ^a^	4.7 ± 2.5 ^a^	5.1 ± 3.3 ^a^
Saliva uptake (g/g)	1.29 ± 0.96 ^a^	1.34 ± 1.4 ^a^	1.12 ± 1.14 ^a^
Bolus particle size < 1.6 mm (%)	79 ± 14 ^a^	82 ± 13 ^a^	78 ± 16 ^a^

M ± SD; different letters mean significant difference between gels at *p* < 0.05.

## Data Availability

The data that support the findings of this study are available from the corresponding author upon reasonable request.
